# High-Resolution Bioprinting of Recombinant Human Collagen Type III

**DOI:** 10.3390/polym13172973

**Published:** 2021-09-01

**Authors:** Rory Gibney, Jennifer Patterson, Eleonora Ferraris

**Affiliations:** 1 Department of Mechanical Engineering, KU Leuven, Campus De Nayer, 2860 Sint-Katelijne-Waver, Belgium; 2 Department of Materials Engineering, KU Leuven, 3001 Leuven, Belgium; 3 Biomaterials and Regenerative Medicine Group, IMDEA Materials Institute, Getafe, 28906 Madrid, Spain

**Keywords:** aerosol jet^®^ printing, collagen, recombinant collagen, bioprinting, cornea

## Abstract

The development of commercial collagen inks for extrusion-based bioprinting has increased the amount of research on pure collagen bioprinting, i.e., collagen inks not mixed with gelatin, alginate, or other more common biomaterial inks. New printing techniques have also improved the resolution achievable with pure collagen bioprinting. However, the resultant collagen constructs still appear too weak to replicate dense collagenous tissues, such as the cornea. This work aims to demonstrate the first reported case of bioprinted recombinant collagen films with suitable optical and mechanical properties for corneal tissue engineering. The printing technology used, aerosol jet^®^ printing (AJP), is a high-resolution printing method normally used to deposit conductive inks for electronic printing. In this work, AJP was employed to deposit recombinant human collagen type III (RHCIII) in overlapping continuous lines of 60 µm to form thin layers. Layers were repeated up to 764 times to result in a construct that was considered a few hundred microns thick when swollen. Samples were subsequently neutralised and crosslinked using EDC:NHS crosslinking. Nanoindentation and absorbance measurements were conducted, and the results show that the AJP-deposited RHCIII samples possess suitable mechanical and optical properties for corneal tissue engineering: an average effective elastic modulus of 506 ± 173 kPa and transparency ≥87% at all visible wavelengths. Circular dichroism showed that there was some loss of helicity of the collagen due to aerosolisation. SDS-PAGE and pepsin digestion were used to show that while some collagen is degraded due to aerosolisation, it remains an inaccessible substrate for pepsin cleavage.

## 1. Introduction

Collagen bioprinting typically suffers from poor resolution, low print fidelity, and results in weak collagen hydrogels (elastic modulus of <10 kPa) [1,2,3,4,5]. Printing techniques, such as embedded extrusion-based bioprinting, have recently improved the resolution that can be achieved in collagen printing [6]. However, the collagen constructs that were printed with this method remained relatively weak (similar to other collagen bioprinting examples) until they were treated with a glutaraldehyde fixation step (evidence in the Supplementary Material of [6]). The typically weak collagen hydrogels that result from most existing bioprinting methods are useful for cell encapsulation, but it is difficult to see them replicating dense collagenous native tissues, such as the cornea, cartilage, and bone. All commercial collagen inks and examples of collagen bioprinting reported so far have used animal-derived collagens, which have potential risks when used in humans (disease transmission, allergens, pathogens, immunogenicity) [7]. Recombinant collagens have received growing interest due to the elimination of the possible risks associated with the use of animal-derived collagens in humans. Recombinant collagens are produced by inserting the relevant gene into a host organism, resulting in a purer product in contrast to extracted collagens and the capability to experiment with the amino acid sequence through gene modification. The host organism selected for recombinant proteins is usually a simple microorganism that is well studied and is easily grown in large quantities within a bioreactor, such as *E. coli* [8] and yeast [9], although plants and animals have also been used [10,11]. However, these simple microorganisms lack the environment, various enzymes, and other proteins that facilitate collagen production and perform post-translational modifications (PTMs). PTMs are very important for collagen stability, and fibril mechanics. A number of forms of osteogenesis imperfecta (OI) are associated with incomplete PTMs [12]. One of the more prevalent PTMs is the hydroxylation of proline residues into hydroxyproline by prolyl hydroxylase; hydroxyproline accounts for 90–150 residues per 1000 in collagen, depending on collagen type [13]. This particular PTM was addressed in part by the co-expression of prolyl 4-hydroxylase, the enzyme that hydroxylates proline residues into 4-hydroxyproline, and COL3A1, the alpha chain that assembles homotrimers to make collagen type III [9]. This co-expression system facilitated the efficient production of hydroxyproline in recombinant human collagen type III (RHCIII) at concentrations similar to that of native collagen type III [9]. This method of production of RHCIII was first commercialised by FibroGen^®^ and was also shown to be effective in producing human collagen type I or II, albeit at lower yields than when used to produce RHCIII [14]. Although it is not clear if other important PTMs were addressed, such as hydroxylation and the subsequent glycosylation of lysine residues, this remains one of the most efficient methods of recombinant collagen production. The use of recombinant collagens has been limited due to their high cost relative to animal-derived or even human collagens; however, this efficient method of production of RHCIII allows RHCIII to be a feasible material for scaffold fabrication.

**Figure 1:**
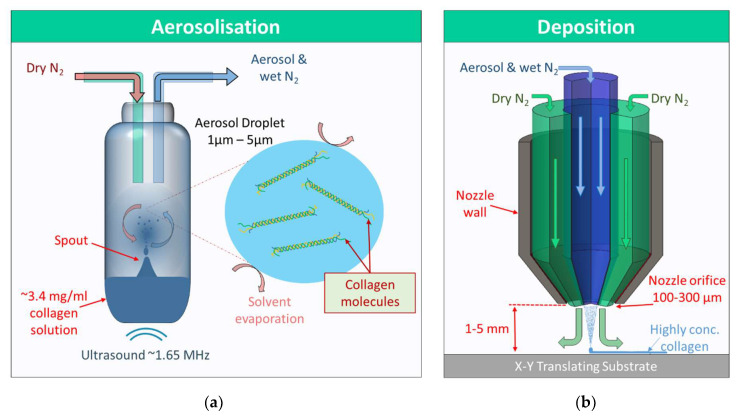
(**a**) Aerosolisation begins with 1.65 MHz ultrasonic waves forming a spout in the vial containing the collagen solution. Aerosol droplets are dispersed from the spout and become entrained in a nitrogen gas flow towards the deposition head. The rate of solvent evaporation is high due to the high surface area to volume ratio of the aerosol droplets and the dry nitrogen gas. (**b**) The aerosol and high humidity N_2_ is collimated by a co-axial flow of nitrogen, which further dries the aerosol droplets and separates them from the walls of the nozzle. The aerosol coalesces on a XY translating substrate forming highly concentrated collagen printed lines.

Aerosol jet^®^ printing (AJP) is a printing technique more commonly associated with printed electronics, but AJP has also been used to deposit biological materials such as DNA, enzymes, and silk fibroin [15,16,17,18]. An AJP system can be viewed as three critical modules: the mass flow controller (MFC) module, which controls the flow of carrier and sheath gas lines (containing nitrogen gas), the atomiser, seen in Figure 1a, which forms the aerosol ultrasonically, and the deposition head, seen in Figure 1b, which aerodynamically focuses the aerosol and directs it towards the substrate. The ultrasonic atomiser forms an aerosol by directing ultrasonic waves from a 1.65 MHz piezoelectric diaphragm through a water bath into a glass vial containing the ink; then, the surface of the ink resonates and forms a spout from which aerosol droplets are scattered. A separate flow of cold water around the piezoelectric circuitry and the water bath body prevents overheating of the transducer and bath, keeping it around 16–18 °C. The aerosol droplets mix with the carrier gas and become entrained in the flow towards the deposition head. In the deposition head, the aerosol droplets are collimated by an annular flow of gas (sheath gas), which separates the aerosol from the walls of the nozzle. The nozzle has an orifice of 100–300 µm in diameter, but the sheath gas allows the deposition of print lines that are a fraction of the nozzle orifice diameter in width, as low as 10 µm [19]. The collimated aerosol droplets continue their path out of the nozzle for up to ≈5 mm, and hence, they can be printed on rough or curved surfaces. Due to the aerosol droplets having a very high surface area to volume ratio, and the absence of moisture in the carrier gas, the rate of solvent evaporation is very high during transit of the aerosol droplets [20,21]. Once coalesced on the print substrate, the solvent evaporation continues at a high rate due to the small geometry of the printed lines and the high air flow from the sheath gas.

When AJP is used to print collagen, the result is a dense collagen film that is optically clear and can be layered hundreds of times to reach a clinically relevant size for corneal substitutes. From previous preliminary research, each layer of AJP-deposited RHCIII was estimated to be around 330 nm thick on average (in swollen state), which was inferred from thickness measurements of 288 layer samples using optical coherence tomography [22]. In general, the thickness of AJP-deposited layers largely depends upon the print parameters (particularly print speed and aerosol flow rate) and the concentration of the ink. It was also known that the viscosity of RHCIII at the concentration at which it is supplied, 3.4 mg/mL, was around the limit of allowable ink viscosity for aerosolisation in the AJP. Given the achievable geometry of AJP and the clarity of AJP-deposited RHCIII, it was deemed of interest in corneal tissue engineering and hence was investigated within the context of a possible corneal stromal substitute.

Corneal stromal substitutes/replacements are intended to be an alternative to corneal transplant where biocompatible materials are used to regenerate an impaired cornea [23]. The cornea’s low metabolic demand and lack of blood vessels facilitates the use of such strategies as a treatment. There is also a reduced risk of rejection due to the cornea’s “immune privilege” [24]. A survey carried out between 2012 and 2013 estimated that approximately 185,000 corneal transplants are performed each year in 116 different countries, and that there are 12.7 million people on corneal transplant waiting lists, but only the needs of one in 70 are covered [25]. Corneal stromal substitutes, along with public awareness campaigns to increase donation rates, could address this donor shortage [26,27].

The corneal stroma is one of five distinct layers in the cornea, which are (from anterior to posterior): the epithelium, Bowman’s Membrane, the stroma, Descemet’s Membrane, and the endothelium [28]. The corneal stroma accounts for up to 90% of the cornea’s thickness and is mostly composed of collagen type I, with significant amounts of collagen type V, proteoglycans, and cells (mostly keratocytes) [28,29]. Although collagen hydrogels can easily be made in the lab, the collagen of the corneal stroma is assembled into fibrils that are much narrower and more uniform than in collagen hydrogels or in other connective tissues. These narrower fibrils scatter less light, allowing the cornea to be highly transparent; light scattering of fibrils has been shown to be related to fibril diameter [30]. The fibrils are heterotypic with collagen type I interweaved with small amounts of collagen type V (around 10%). This interweaving is believed to regulate the fibril diameter along with other factors, such as the electrostatic repulsion between fibrils, which is generated, in part, by chloride ions forming complexes with fibrils [31]. The corneal fibrils are aligned in parallel into lamellae that have been measured in the range of 1–200 µm wide and 0.2–2.5 µm thick [32]. The lamellae can span the entire cornea and tend to be oriented orthogonal to each other in the central stroma, whereas in the peripheral stroma, they are oriented circumferentially [28].

RHCIII has been made into highly transparent corneal substitutes using contact lens molds in some pioneering work by May Griffith et al. [33,34]. These went through a 4-year clinical trial with 10 patients and no cases of rejection [35]. In order to achieve the required transmissibility for the stromal substitute, they limited the extent of fibrillogenesis in their RHCIII hydrogels. Native type fibrils begin to form within the pH range of 5.0–8.5, ionic strength between 0.1 and 0.8 [36], and temperatures from 4 to 37 °C [37], and experimenting with these conditions can elicit different fibrillar architectures. The RHCIII gels were formed at a relatively low pH 5.5, a mid-range ionic strength, and EDC:NHS crosslinking was simultaneously employed during gelation to improve mechanical strength. Temperature was also a factor, as the components were initially mixed on ice, then incubated in a room temperature for 24 h, and finally incubated at 37 °C for 24 h to complete curing [33]. Under these conditions, sparse narrow atypical collagen fibrils were formed that allowed the RHCIII gel to be highly transparent [38]. However, the mechanical properties of the RHCIII gels left them unsuitable for suturing; hence, they were implanted with overlying mattress sutures anchored around the periphery of the resected site. These mattress sutures had a negative impact on the surface morphology of the gels, making them rough and the vision of the recipient poor. Aerosol jet printing of RHCIII could improve on the achievements of the RHCIII corneal substitutes by making it patient-specific, and the high resolution of AJP could help replicate the structure of the native cornea better. We have previously reported on the suitability of the use of AJP RHCIII constructs for the culture of human corneal mesenchymal stem cells, where they were seen to proliferate and penetrate into the scaffold [39]. However, the aerosolisation process exposes the RHCIII to a number of environmental extremes, including high shear, and possible extremes of temperature and pressure via sonication. The aim of this study was to characterise the suitability of AJP for the production of RHCIII hydrogels with suitable optical and physical properties for corneal tissue engineering, and to characterise the viscosity of the RHCIII ink used and any degradation or denaturation caused by the exposure to intense ultrasound and aerosolization in the AJP process.

## 2. Materials and Methods

### 2.1. Materials

First, 3.4 mg/mL recombinant human collagen type III (RHCIII, produced by yeast; *pichia pastoris*) solutions in 0.01 M hydrochloric were procured from MyBioSource (San Diego, CA, USA) and later Abcam (Abcam (Netherlands) B.V.) after the production of RHCIII was ceased at MyBioSource. The α-1 chains that assemble into a trimer to make RHCIII had an expected molecular weight of 138.6 kDa based on the UniProt accession P02461 sequence. EDC (N-(3-Dimethylaminopropyl)-N′-ethylcarbodiimide hydrochloride), hexamethyldisiloxane (HMDS), and the ProteoSilver staining kit were purchased from Sigma Aldrich (Leuven, Belgium), and N-hydroxysuccinimide (NHS) was purchased from Fluka (Buchs, Switzerland). All other materials were purchased from VWR Belgium.

### 2.2. Production of Aerosol Jet-Printed RHCIII Samples

#### 2.2.1. Aerosol Jet Printing (AJP) of RHCIII Samples

An Optomec^®^ AJ300 was used to print two sets of circular samples: twelve 4.5 mm diameter samples (B1–B12) and eight 7 mm diameter samples (A1–A8). The number of layers and print time for each set of samples were 764 layers in 4 h and 384 layers in 8–9 h, respectively. The 7 mm samples were intended to fully cover the well of a 96-well plate for transmission measurements. The sample diameter was reduced to 4.5 mm for samples that were not to proceed to transmission measurements in order to achieve thicker samples in a shorter print time. The toolpath generator normally used on the AJ300 produced code was very computationally heavy and made the system liable to crash when compiling or executing toolpaths for samples with many layers. Hence, a MATLAB^®^ program was developed that could generate toolpath programs for rectilinear rasters of user-defined circles with user-defined space between print lines (normally 60 µm). The program rotated each even-numbered layer by 90° relative to the preceding layer, whereas each odd-numbered layer was rotated by a user-defined number of degrees, typically 15°, since this led to samples that appeared to possess a smooth and flat topography. All samples were printed using a nozzle with an orifice diameter of 100 µm, print speed of 11.5 mm/s, sheath flow of 60 cc/min, atomiser flow of 40 cc/min, and atomiser power of 90% with slight variations in atomiser power and flows to account for low or high atomisation. It was known from experience that printing with these parameter values and with this many layers leads to the growth of the sample diameter of about 0.5 mm. Hence, the programmed diameters were 4 mm and 6.5 mm for the 4.5 mm and 7 mm samples, respectively. All 4.5 mm samples were measured after printing to confirm that this was a precise and repeatable deviation that could be incorporated into the toolpath program generator. For all samples, 850 µL were pipetted into the vial at the beginning of a print, and this volume was topped up as needed, every 1–1.5 h of printing. Once the samples were printed, they were kept dry at 4 °C until the entire sample set was printed; then, they were observed under a microscope before proceeding to crosslinking.

#### 2.2.2. Crosslinking of Aerosol Jet Printed RHCIII

EDC:NHS crosslinking was performed in a co-solvent made of 2 parts: an aqueous 0.01 M 2-(N-morpholino)ethanesulfonic acid (MES buffer) solution and 3 parts ethanol. The pH of the MES buffer was adjusted to pH 8–9 using 1 M NaOH. Then, the MES buffer was added slowly to the ethanol to prevent precipitation of the MES, and the solution was left mixing for ≈1 h. The pH was checked at this point, and further adjustment of the pH (to pH 8–9) was performed if necessary. Then, EDC and NHS were dissolved in the reaction buffer at a 1:1 molar ratio, final concentration 40 µg/mL EDC, 24 µg/mL NHS. Then, 1 mL of the crosslinking solution was carefully pipetted into each well in a 24-well plate containing the samples on ice. Then, the well plate was stored at 4 °C for 18 h. After 18 h, the crosslinking solution was removed from the wells, and the samples were rinsed twice in ultrapure water and stored in 1X PBS @ 4 °C.

### 2.3. Optical Characterisation

The optical properties of the 7 mm samples were measured using a plate reader. The samples were swollen in 1X PBS, detached from their substrate, and placed in a flat-bottomed 96-well plate with 200 µL of 1X PBS. The samples were made slightly wider than the wells to ensure full well coverage. A corneal scalpel and tweezers were used to ensure the sample was flat and completely covering the bottom of the well. The absorbance was measured with wells containing only 1X PBS as blanks. The absorbance was measured between 390 and 700 nm and converted to transmission percentage, using the Beer–Lambert law equation for transmission:

(1) $$Transmission=10^{-Absorbance}.$$

The refractive index was measured using an Atago 7000X. The samples were swollen in 1X PBS and then placed on the prism of the refractometer; each measurement was repeated three times.

### 2.4. Physical Characterisation

#### 2.4.1. Nanoindentation

The mechanical properties of the AJP samples were characterised using a Chiaro nanoindenter (Optics11). This system and its sister system, the Piuma, are emerging tools for measuring the mechanical properties of hydrogels and for mechanobiology [40,41,42]. Both are comprised of a spherical indenter on a cantilever of known stiffness, the deflection of which is measured by interferometry via a ferrule-topped optical fiber [43]. A piezoelectric actuator displaces the probe by a user-defined distance over a user-defined period of time (the indentation profile in Figure 3a) leading to deflection of the cantilever. Then, the deflection is used to calculate the load and the stiffness thereafter. While these systems are mostly used to measure local variations in stiffness, they are also used to measure the bulk modulus of hydrogels [44,45,46]. This form of micro/nano-indentation has provided results consistent with tensile and compressive test results [47]. For these measurements, a probe with a cantilever stiffness of 5.2 N/m and a 50 µm diameter spherical indenter was selected. A total of 25 indentations were performed on each sample in a 5 × 5 matrix with 200 µm spacing between indentations in both directions. The indentation profile went to a maximum of 12 µm at an indentation rate of ≈1.7 µm/s. An 8 µm offset was included for all measurements to ensure that the probe was not in contact with the sample surface upon beginning the indentation. The effective elastic modulus was calculated by the Chiaro software using the slope of the loading curve according to a Hertz model. Only samples that remained attached to their substrate could be measured accurately. All measurements were taken in PBS with samples that had been equilibrated in PBS overnight.

#### 2.4.2. Scanning Electron Microscopy (SEM)

Sample preparation was performed according to a protocol that was a modified version of that reported by Raub et al. [48]. Briefly, samples were fixed in 4% glutaraldehyde in 1X PBS for 1 h at room temperature, then washed 3 times in PBS for 7 min and 2 times in ddH_2_O for 7 min. Then, samples were dehydrated in increasing concentrations of ethanol in ddH_2_O (30%, 50%, 70%, 90%, and 100% *v*/*v*) for 10 min each and twice in 100% ethanol. The samples were further dehydrated in increasing concentrations of HMDS in ethanol (33%, 50%, 66%, and 100% *v*/*v*) for 15 min each and twice in 100%. Then, samples were torn apart with tweezers if possible or cut and left to dry on aluminium foil under a fume hood overnight. The dried samples were mounted on SEM stubs using carbon tape and sputter-coated with platinum to a thickness of 5 nm. The samples were imaged at 5 kV.

#### 2.4.3. Swelling Ratio and Water Content

Both the swelling ratio and water content of the scaffolds were calculated to represent the swelling properties of the scaffold, and for use in comparisons to the native cornea for which water content is frequently reported. In order to measure the swelling ratio and water content, six of the 4.5 mm AJP RHCIII samples were incubated in 1X PBS at 4 °C overnight. The wet samples were turned over quickly on a filter paper to remove the excess water and then weighed. Then, the samples were rinsed twice in ultrapure water and frozen. The frozen samples were lyophiliised and weighed to measure the dry weight. The swelling ratio was calculated using Equation (2) and the water content was calculated using Equation (3); both are shown below:

(2) $$\mathrm{Swelling}\;\mathrm{ratio}=\frac{wet\;weight-dry\;weight}{dry\;weight}$$

(3) $$\mathrm{Water}\;\mathrm{content}=\frac{wet\;weight-dry\;weight}{wet\;weight}\times 100\%.$$

### 2.5. AJP RHCIII Characterisation

#### 2.5.1. Viscosity

Viscosity measurements were made using a Lovis 2000 Rolling-Ball Viscometer (Anton Parr, Graz, Austria). Measurements were made at 20 °C, 25 °C, 30 °C, 35 °C, and 40 °C. A standard size capillary tube was filled carefully, avoiding bubbles, with 600 µL of RHCIII using a syringe and a filling adapter. A ball dispenser was employed to drop a 1.8 mm inert gold ball into the capillary tube. The tube was checked for bubbles before being sealed and fixed into the viscometer. The viscometer performed a measurement by measuring the time for the ball to fall through the liquid several times in opposing directions; then, data from these runs were used to calculate the Lovis Dynamic Viscosity, µ.

#### 2.5.2. Sodium Dodecyl Sulfate Polyacrylamide Gel Electrophoresis (SDS-PAGE)

Samples were taken from the printer during operation in two differet printer set-ups designed to indicate whether any detected degradation was a result of ultrasonic atomisation or solely due to the exposure of the RHCIII to 1.65 MHz ultrasound. To measure degradation as a result of ultrasonic atomisation, the printing apparatus was set up similar as if to print but without deposition. Aliquots of 850 µL RHCIII solution were pipetted into a vial and subjected to aerosolisation for up to 2 h at the minimum atomiser power that generated sufficient aerosol for printing (90%). The sheath flow was set at 100 cc/min to simulate the pressure in the atomiser during printing (0.4–0.5 bar). To measure degradation as a result of exposure to 1.65 MHz ultrasound alone, a small amount of RHCIII solution (150 µL), which was too low to result in atomisation, was pipetted into a vial. The vial was closed tightly with a cap to prevent evaporation and placed in the atomiser at 90% power for up to 2 h. In each case, samples were taken from the vial at 20, 60, and 120 min. For comparison, printed samples, 4.5 mm in diameter and 75 layers thick, were analysed by re-solubilising them in ≈100 µL 0.01 M HCl and diluting them appropriately. Samples were also subjected to a pepsin digestion (0.2 mg/mL pepsin at room temperature for 1.5 h) to remove any non-helical collagen. SDS-PAGE was performed using a Mini Gel Tank, the Invitrogen NuPAGE^®^ Electophoresis System (ThermoFisher Scientific) under reducing conditions in order to reduce the disulphide bonds in the C-termini of the alpha-1 chains. A ProteoSilver silver staining kit (Sigma Aldrich) was used to visualise the proteins on the gels.

#### 2.5.3. Circular Dichroism (CD)

The CD spectra of untreated RHCIII (control) and RHCIII that had been treated with aerosolisation for 120 min, similar to the sample treatment for SDS-PAGE, were measured using a Jasco J-1500-150ST CD spectrometer. The spectra were measured between 190 and 260 nm, with a scan speed of 50 nm/min and wavelength interval of 0.5 nm. Rectangular quartz cuvettes with a 1 mm path length were first measured with 0.01 M hydrochloric acid to obtain the background spectra, which was subtracted from the results. The treated RHCIII sample and the control were each diluted to 0.3 mg/mL for the measurements, using 0.01 M HCl.

## 3. Results

### 3.1. Aerosol Jet-Printed Recombinant Human Collagen Type III (AJP RHCIII) Sample Production

**Figure 2:**
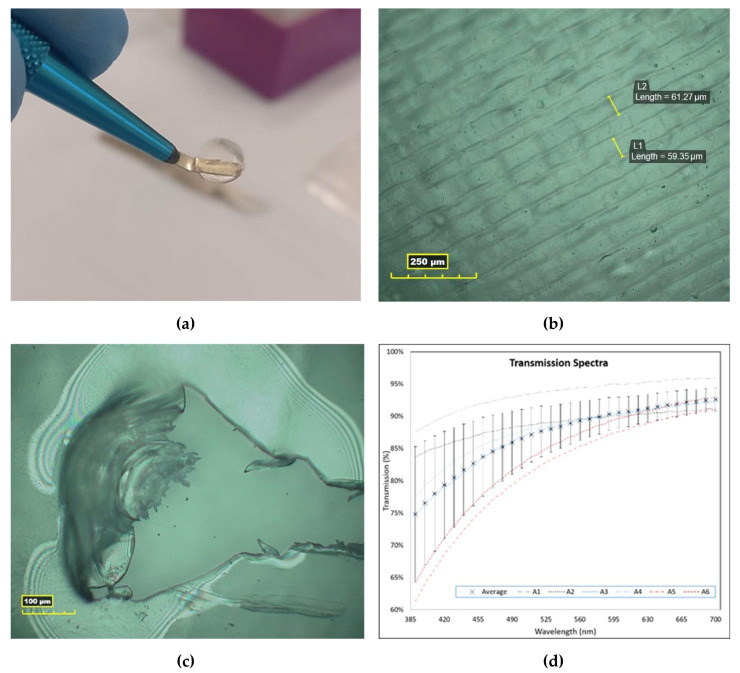
(**a**) A crosslinked 7 mm sample swollen in PBS held on a corneal scalpel; (**b**) print lines ≈60 µm in width in the centre of a 7 mm sample imaged under microscope after printing; (**c**) a failed sample in which a hole was torn to exhibit the birefringence seen in the samples. (**d**) The transmission spectra of each of the 7 mm samples measured, cross points with error bars represent the average transmission and standard deviation at 10 nm intervals.

All samples were observed under a microscope after sample production, and their diameters were measured optically. The 4.5 mm samples had an average diameter of 4.54 mm with a standard deviation (SD) of 0.03 mm, and the 7 mm samples had an average diameter of 7.06 mm with a standard deviation of 0.04 mm. Samples were on average 0.55 mm ± 0.035 mm (± the SD) larger than their programmed diameters (4 mm and 6.5 mm), but as mentioned previously, this was expected. Print lines were difficult to resolve due to the transparency of the material and flatness of the printed lines but some print lines, 60 µm wide, could be resolved in the centre of samples, as seen in Figure 2b. The samples were labelled A(x) (7 mm sample) or B(x) (4.5 mm sample) with ‘x’ denoting the sequence of their print.

### 3.2. Optical Characterisation

The samples appeared highly transparent to the eye, as seen in Figure 2a, and this was confirmed in the transmission results, which measured an average transmission of 87.5 ± 6% (± the SD) for the 7 mm samples (A1–A6) over all visible wavelengths measured. Samples transmitted less light at wavelengths below 450 nm, as seen in Figure 2d. Two samples, A5 and A6, exhibited lower transmission spectra than the other samples, which was noticeable upon close inspection by eye. It was likely caused by an increased presence of fibrils in the samples due to hydration of the sample during crosslinking. The average refractive index (RI) of the samples was 1.3463 ± 0.0223 (± the SD).

### 3.3. Physical Characterisation

#### 3.3.1. Nano-Indentation

**Figure 3:**
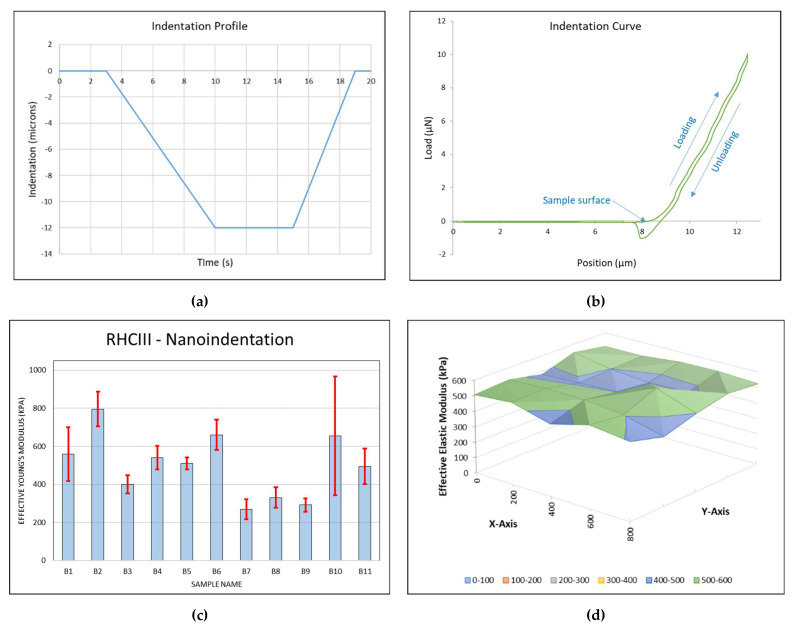
(**a**) The indentation profile used in the study which included an 8 µm off-set, so the sample was indented 4 µm. (**b**) The indentation curve illustrates the offset, with the load only increasing after the probe has moved 8 µm. The loading slope of the loading curve is used to calculate the elastic modulus. (**c**) The average effective E of each sample from 25 indentations with error bars representing the standard deviation. (**d**) An effective E map of sample B5 representing the 800 µm × 800 µm 25 point indentation matrix with 200 µm between indentations.

The load–indentation curve shown in Figure 3b was typical of the 25 indentations performed on each sample, indicating quite an elastic response with low hyteresis observed in the unloading curve. The negative load at the end of the unloading curve, which was common amongst all indentations, was likely due to the probe tip sticking to the sample surface briefly. The average effective elastic modulus found for the 4.5 mm diameter samples (B1–B11) measured was 506 ± 173 kPa (± the SD). A twelfth sample (B12) could not be measured since the sample had detached from its substrate. The effective elastic modulus of each sample can be seen in Figure 3c. Local variations of the effective elastic modulus tended to be relatively small, as in Figure 3d, although some very stiff indentations lead to higher standard deviations in some samples, particularly in sample B10, which recorded one outlier indentation measuring an effective elastic modulus of 2.2 MPa. The effective elastic modulus was reported, since Poisson’s ratio was unknown for the material.

#### 3.3.2. Swelling Ratio

The mean wet weight of the 4.5 mm samples was 4.9 ± 0.7 mg (± the SD), and the mean dry weight after lyophilisation was 0.66 ± 0.16 mg (± the SD). The mean swelling ratio was calculated as 6.8 ± 1.6 (± the SD), and the mean water content was calculated to be 86.7 ± 2.6% (± the SD).

#### 3.3.3. Scanning Electron Microscopy (SEM)

**Figure 4:**
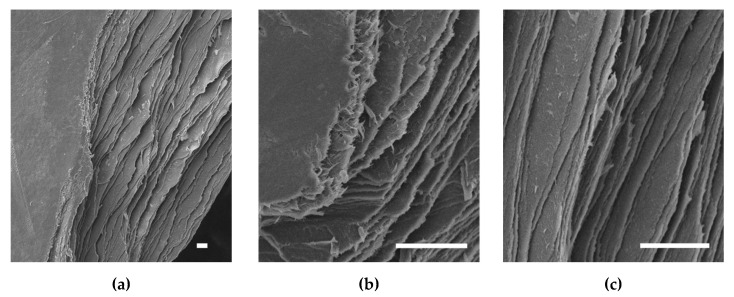
SEM images of 4.5 mm AJP RHCIII samples at the edges where they were torn apart, illustrating the distinct layers from the printing process. (**a**) Full section view showing smooth sample surface and layers beneath. Higher magnification images showing, (**b**), fibrous structures at observed at tear-edges, and (**c**), density of the layers at the sections. All scale bars represent 10 µm.

Whilst no pores could be resolved on the top or bottom surfaces of the samples, clearly defined distinct layers could be seen at the edges where samples were torn apart, as seen in Figure 4. These layers continued across the entire span of the samples, from what could be seen at the cross-sections, and hence were deemed to be the printed layers. The thickness of the layers was too thin to be accurately measured in SEM. Fibrous structures seen at tear edges could not be clearly resolved and would require TEM or AFM to determine any D-banded patterns, if present.

### 3.4. AJP RHCIII Characterisation

#### 3.4.1. Viscosity

A viscosity of 18.2 mPa·s was measured for RHCIII at 20 °C, which is above the recommended viscosity range of the Optomec ultrasonic atomiser: ≤5 mPa·s. However, this did not prevent aerosolisation. In this soluble form, the viscosity of the RHCIII decreased with increase in temperature as expected. The viscosities for 25 °C, 30 °C, and 35 °C were 15.9, 12.2, and 3.0 mPa·s, respectively. A value could not be resolved at 40 °C, as the viscosity was too low.

#### 3.4.2. SDS-PAGE

**Figure 5:**
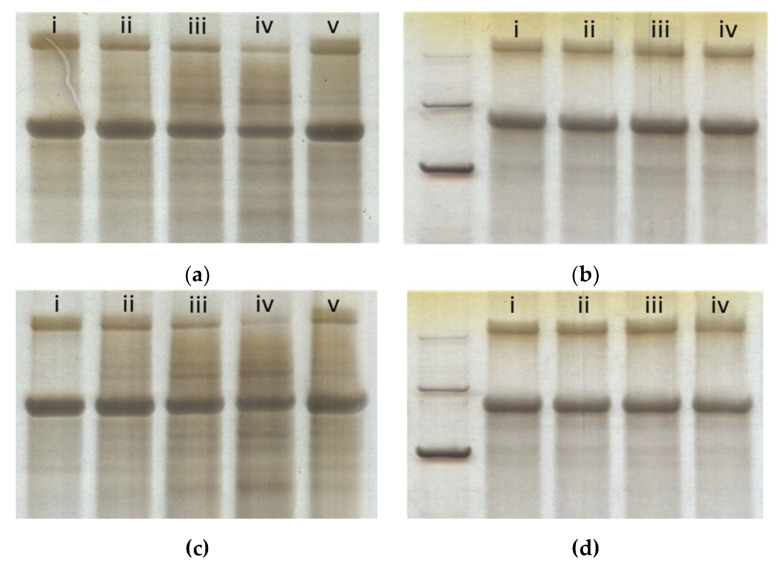
SDS-PAGE gels of ultrasonically atomised RHCIII, (**a**,**c**), and non-atomised RHCIII that was exposed to 1.65 MHz ultrasound, (**b**,**d**). Samples in gels (**c**,**d**) were digested with pepsin, whereas gels (**a**,**b**) are their non-digested controls. All lanes marked (i)–(iv) correspond to 0, 20, 60, and 120 min. Lanes marked (v) were re-solubilised printed samples. Molecular weight standards in the left-most lane of (**b**,**d**) mark 250, 150, and a high-intensity band at 100 kDa.

SDS-PAGE analysis showed that some RHCIII was gradually degraded from the exposure to ultrasonic atomisation, but a clear α-1 band remained at the expected molecular weight of ≈130–140 kDa. β-bands at the top of the gel showed that not all disulphide bonds were reduced. It appeared that the degradation was the result of the shear stresses involved in atomisation rather than the exposure to 1.65 MHz ultrasound alone, since no degradation could be seen in the samples that were not atomised; see Figure 5b,d. Printed samples that were re-solubilised did not exhibit any evidence of an additional mode of degradation due to deposition. Lanes resolved for the printed samples appeared similar to those for the 20- and 60-min sample, which corresponded to the approximate print time of these printed samples, ≈40 min. The degraded collagen showed no preferential molecular weight, although bands did seem to be emerging around 100 kDa and 80 kDa, after 120 min of aerosolisation; see lane iv Figure 5a,c. The pepsin-digested samples in Figure 5c,d appeared very similar to their non-digested counterparts. Although there did seem to be slightly increased non-specific staining in Figure 5c, the same level of band depletion with time was seen. The pepsin digestion did not deplete the staining of the α-1 bands nor the emerging bands at 100 and 80 kDa, suggesting that the degraded collagen remained in some conformation that made it inaccessible as a substrate for cleavage, i.e., a triple helix conformation.

#### 3.4.3. Circular Dichroism Spectra

**Figure 6:**
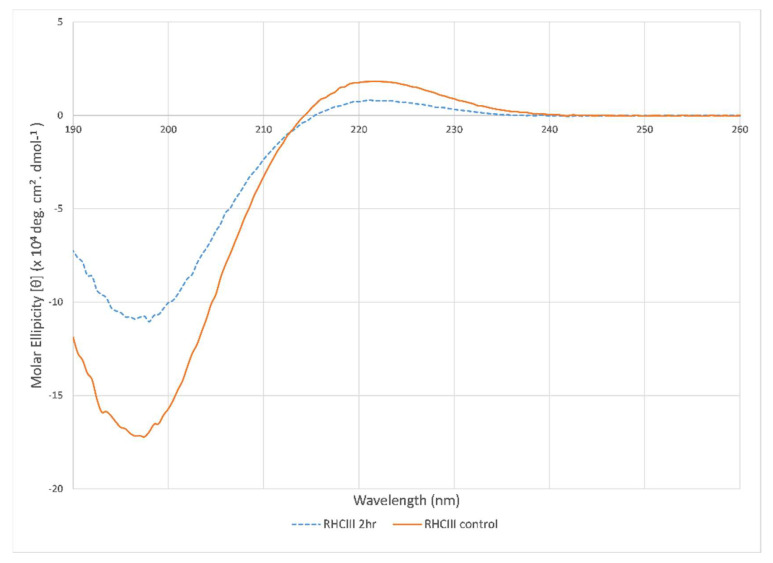
CD spectra of untreated RHCIII and 2-h sonicated RHCIII showing the characteristic collagen CD ellipticity with a positive peak centered on 221 nm and a negative peak centered on 197 nm.

Typical collagen CD spectra were measured for both sonicated and untreated control RHCIII samples with a minimum ellipticity peak centered at 198 nm and a maximum ellipticity centered at 221 nm (Figure 6). However, a significant drop in peak intensities for the 2-h treated sample indicates denaturation of the collagen due to the exposure to ultrasonic atomisation. There was also a red-shift in the cross-over point from 214.7 to 215.6 nm; this was likely due to contributions from randomly coiled collagen and collagen degradants, which would contribute a negative ellipticity at these wavelengths [49].

## 4. Discussion

This work is the first research article on aerosol jet printing of collagen and the first research article on 3D-printed recombinant human collagen; only collagen-like and collagen-based recombinant proteins have been printed previously [50]. Sample printing has gone through a series of phases to achieve AJP RHCIII samples of this size and the number of layers within a print time that allows multiple samples to be printed in one day. In this context, these samples are near the limit of what can be achieved with RHCIII on the Optomec^®^ AJ300 AJP system used in this study, and near-constant observation is required to monitor the amount of aerosol generated and the deposition as well as make changes to the flow rates when necessary. The high flow rates used lead to a high relative pressure in the deposition head (0.4–0.5 bar), compared to normal operating conditions for AJP (0–0.2 bar). It appeared that this, along with the high print speed, was likely a major contributing factor to the increased sample diameter, as the resultant excessive jet could often be seen to push some material over the edge of the deposited area during turns. Nevertheless, it has been shown here that this “non-programmed deposition” is precise and repeatable and hence can be incorporated into the MATLAB^®^ toolpath generator. A more concentrated ink may allow quicker sample production with less need for high flows and print speeds; however, a more concentrated RHCIII solution would likely be too viscous to atomise. Along with the high print fidelity measured, very little contraction due to crosslinking was observed in both sample sets, which was likely due to the relatively low EDC concentration and the density of the material.

The AJP process, more specifically ultrasonic atomisation, leads to some denaturation and degradation of the RHCIII. However, this happens gradually, and fresh RHCIII was added to the vial during a print run every hour as the RHCIII was consumed; therefore, the amount of degraded/denatured collagen is not likely to reach the levels seen in the 2-h treatment SDS-PAGE and CD spectra measurements. The fact that the degradation and denaturation of the RHCIII occurs gradually means that the construct will not be homogenous and that the layers around the hour mark, before the RHCIII is replenished, will contain more denatured/degraded RHCIII than the preceding layers, and the first layers will contain little to no denatured/degraded RHCIII. However, the results indicate that there should be no single layer in any of the samples where degraded RHCIII is in the majority, and intact RHCIII accounts for the bulk of material at all times in this study. It was a concern that the RHCIII and any fragments in random coil formations may absorb more light, but the transmission study has shown that this AJP RHCIII, which contains some degraded/denatured collagen, is highly transmissible. The lack of a difference between the pepsin-digested AJP-treated RHCIII and their controls indicates that very little if any of the degraded collagen may be in random coil formation. A high concentration of pepsin (0.2 mg/mL) was used for the pepsin digestion after similar results were seen in preliminary experiments with lower pepsin concentrations (0.04 mg/mL). Further experiments will be required to investigate if the lower magnitude CD spectra observed for the 2-h sonicated RHCIII could be due to the presence of fragmented collagen that remains in a triple helix conformation rather than a random coil conformation. It is not clear if the inclusion of degraded RHCIII will have any impact on the response of corneal cells to the potential scaffold. In cartilage, the presence of denatured and degraded collagen in culture media has been shown to upregulate the expression of matrix metalloproteinases (MMPs) in chondrocytes [51,52]. If these results would be replicated for fragmented collagen in the scaffold material with corneal cells, it could lead to faster remodeling or deterioration of the construct.

The birefringence exhibited by samples, seen in Figure 2c, suggests that the RHCIII is in liquid crystal conformations within the AJP constructs, although further work with cross-polarised microscopy of sample sections is required to confirm this. This could reveal similarities between the AJP-deposited RHCIII and the liquid crystal collagen type I films produced by Giraud-Guille, who performed many investigations into the liquid crystallinity of highly concentrated collagen type I films made from solutions that had been sonicated in a cooled ultrasonic bath (20 kHz, 50 W) prior to being concentrated and forming films [53,54,55,56]. It was concluded in those studies that the presence of the fragmented collagen facilitated a quicker formation of liquid crystalline collagen.

The optical characterisation results show that AJP-deposited RHCIII, at least in optical characteristics, is a suitable candidate for a bioprinted cornea, with >80% light at all visible wavelengths transmitted in every sample, similar to the native cornea [29]. Thicker fibrils are likely to be the cause of the higher absorbance in samples A5 and A6. The RI measurement was similarly skewed by one sample with a much higher RI than the other samples. Nevertheless, even the least transparent samples were still within the range of what is considered acceptable for a corneal substitute, and high blue light absorption in the cornea is necessary to protect the retina [57,58]. Despite the efforts to limit fibrillogenesis, thicker light-scattering fibrils could form when the AJP-deposited RHCIII is hydrated for neutralisation and crosslinking, particularly if the pH becomes acidic. The co-solvent used as a crosslinking buffer for the EDC:NHS crosslinking was developed as a medium for crosslinking in earlier work (see Supplementary Material). This crosslinking buffer was determined to best preserve sample morphology and lead to little or no signs of dissolution of the AJP-deposited RHCIII. Ethanol was employed as a co-solvent to slow the hydration of the AJP-deposited RHCIII, which was based on observations reported of collagen’s poor absorption of ethanol [59]. The pH of the crosslinking buffer was adjusted to compensate for the small amount of acid that leached out of the AJP RHCIII upon re-hydration. PBS was purposely excluded from the crosslinking buffer, since phosphate ions are known to have a major influence on collagen precipitation [60]. However, the storage in PBS after crosslinking may still affect the AJP RHCIII if it is not completely neutralised in the crosslinking.

It is difficult to propose any particular fibril assembly for the arrangement of collagen molecules in the AJP-deposited RHCIII, but there is evidence in the SEM images that the collagen retains distinct printed layers after crosslinking and hence does not experience major re-solubilisation during neutralisation and crosslinking, which would be necessary for native-type fibrils to form. Therefore, any fibrils that form are most likely to be atypical very skinny fibrils that assemble within a printed layer. Each printed layer was composed of overlapping printed lines 60 µm in width, as seen in Figure 2b, but these were lost in the sample preparation for SEM. The dense collagenous nature of these layers, and the fact that their estimated thickness (≈330 nm [22]) is within the range of reported corneal stroma lamellae thickness (0.2–2.5 µm [32]), could be considered to be biomimesis of the lamellae of the corneal stroma. This could be enhanced by printing 200 µm wide strips, instead of whole layers, traversing and circumnavigating the centre of the sample for closer mimesis of the corneal lamellae structure [28]. The swelling ratio measurements show that the constructs absorb aqueous media well, which could be advantageous for cell culture but may be a concern for dimensional accuracy should this translate into volumetric swelling. However, this was anticipated. As stated earlier, layers are expected to be ≈330 nm thick when swollen. The transparency of the material made any accurate swollen geometrical measurements unfeasible with the available equipment; however, samples were observed to swell mostly vertically, with no major changes apparent in the sample diameter. The water content calculated for the samples, 86.7%, is between that normally found for the native cornea, ≈78%, and that reported for the RHCIII biosynthetic corneas of May Griffith, ≈90% [38,61]. This could potentially change with cell culture, or with remodelling upon implantation, and the subsequent deposition of proteoglycans, new collagen, and other matrix material.

The elastic moduli measured for the aerosol jet-printed RHCIII samples was far higher than the previously reported elastic moduli results for pure printed collagen found in the literature. The highest reported elastic moduli for printed collagen found were compressive moduli of around 110 kPa for glutaraldehyde-crosslinked bovine collagen type I [6], 47.2 ± 14.2 kPa for EDC-crosslinked porcine-hide collagen, and 21.5 ± 1.4 kPa for porcine-tendon collagen type I, although higher elastic moduli have been reported for constructs made with collagen hybrid inks [62,63]. The aerosol jet-printed RHCIII samples outperformed previous reported attempts of cornea printing both in terms of mechanical and optical properties [64,65,66]. However, moulded collagen hydrogels for the cornea have been reported with higher elastic moduli than the aerosol jet-printed RHCIII samples, although their optical properties were very similar [35,67]. Corneal elasticity has been measured using a number of methods including in vivo methods, such as the Orssengo–Pye method [68], and in vitro methods, such as membrane pressure, strip extensiometry, and nanoindentation, resulting in quite a wide range of reported human corneal Young’s moduli from 0.075 to 20 MPa [69,70,71,72]. The elastic moduli of each of the AJP RHCIII samples fits well within this range and fits within the range of the reported elastic moduli for the human anterior cornea stroma, which was measured using nanoindentation (range: 59–765 kPa, average: 281 ± 214 kPa) [73]. Larger samples would be required to further investigate the mechanical properties of the AJP RHCIII with more established methods for the characterisation of mechanical properties, such as tensile testing. However, a more revealing test of the suitability of AJP RHCIII’s mechanical properties would likely be a test of the suturability of the samples. Suturability has been shown to be a limitation of the moulded RHCIII corneal substitute [74], and from what can be seen from the nanoindentation results and the handling of the AJP RHCIII samples, they appear to have suitable flexibility to allow it to be pierced with a needle. Suturability will likely be tested using samples that are more cornea-shaped (when they are developed) on a rabbit eye.

## 5. Conclusions

Aerosol jet-printed recombinant human collagen type III exhibits higher print resolution, print fidelity, and mechanical strength than any pure collagen bioprinting previously reported. This work demonstrates that aerosol jet printing offers a way of processing pure collagen solutions into dense stiff material to replicate native dense collagenous tissue such as the cornea.

## Data Availability

The data presented in this study are available on request from the corresponding author.

## Supplementary Materials

The following are available online at https://www.mdpi.com/article/10.3390/polym13172973/s1, crosslinking of aerosol jet printed RHCIII, Table S1: Conditions used to investigate the influence of ethanol concentration, pH, and temperature on AJP RHCIII dissolution; Figure S1: Images of samples before and after crosslinking with conditions, and sample ratings which were given according to the prevalence of signs of dissolution, Table S2: Table of the crosslinking performance ratings.
