# Supplementary material for: High-Resolution Bioprinting of Recombinant Human Collagen Type III
Source: Polymers (Basel). 2021 Sep 1;13(17):2973. doi: 10.3390/polym13172973 (PMC8434404; doi:10.3390/polym13172973)
Supplement: Supplementary file 1 [file polymers-13-02973-s001.zip › polymers-1356173-supplementary.pdf]

Supplementary Material

High resolution bioprinting of recombinant human collagen type III

Rory Gibney [1, 2], Jennifer Patterson [2, 3]* and Eleonora Ferraris [1]*

[1] Department of Mechanical Engineering, KU Leuven, Campus De Nayer, Sint-Katelijne-Waver, 2680 Belgium
[2] Department of Materials Engineering, KU Leuven, Leuven, 3001 Belgium
[3] Biomaterials and Regenerative Medicine Group, IMDEA Materials Institute, Getafe, Madrid, Spain 28906

Crosslinking of aerosol jet printed RHCIII

Materials & Methods

Sample production

Samples were printed onto glass coverslips using AJP with a sample diameter of 4 mm and 30 layers. Layers were rectilinear rasters of a circle with line width of 60 µm, each layer was rotated 90° with respect to the orientation of the previous layer. Samples were printed using a 100 µm nozzle at a print speed of 5 mm/s, an aerosol flow rate of 20 cc/min, and a sheath flow rate of 16 cc/min. The samples were observed under the microscope after printing for later comparison.

Crosslinking

To make the buffer, first a 0.01 M solution of MES was made. A 1 M solution of sodium hydroxide was used to adjust the pH of the buffer to either 5.5 or 8.5. The buffers were then added, in suitable amounts, slowly to ethanol while stirring, resulting in 4 different solutions, 40% ethanol pH 5.5, 40% ethanol pH 8.5, 70% ethanol pH 5.5, and 70% ethanol pH 8.5. A 0.4 mg/ml EDC, 0.24 mg/ml NHS solution was formed with each of the 4 buffers. 40 µl of each solution was pipetted onto 2 samples each either at room temperature or on ice. A small volume was used so that the solution could be limited to the coverslip. The wellplates containing the samples were then left at room temperature or in a fridge at 4°C for 24 hours. The crosslinking solution was then removed, and the samples were washed once in cold PBS and twice in cold ddH_2O for 15 minutes. Control samples using the same EDC concentration in 100% ethanol at 4°C and 20°C, and in 0.625 M MES adjusted to pH 5.5 at 37°C (same conditions as Liu et al. 2008)

Condition	Ethanol % v/v	pH
**pH**	5.5	8.5
**Ethanol %**	40%	70%
**Temperature**	4°C	20°C

Table S1: Conditions used to investigate the influence of ethanol concentration, pH, and temperature on AJP RHCIII dissolution.

Crosslinking Performance

Samples were observed by optical microscope after crosslinking and the samples were rated from 1 to 10 based on how closely they resembled the microscope images taken pre-crosslinking. Samples were rated 10 if no difference could be discerned, and 1 if the sample had completely dissolved.

Results & Discussion

Examples of samples ratings can be seen in figure 1, and a table of the ratings is in table 2. The samples crosslinked in 70% ethanol clearly performed better than those at other ethanol concentrations. Despite the high ethanol concentration, clear signs of dissolution could be seen in

40% ethanol pH 5.5 samples compared to 40% ethanol pH 8.5 samples. Some difference was also seen between the different pH in 70% samples, though at a lesser extent. The 100% ethanol samples resulted the survival of surface features but with clear loss of much of the sample thickness. This was expected based on the reported low ethanol absorption exhibited by collagen. The severe thinning seen can be explained by only the sample surface initially being crosslinked, leaving the bulk collagen in the sample in soluble form. The bulk collagen then dissociates out of the sample during the washing steps in aqueous media leaving a thin exoskeleton of the sample behind. Differences in crosslinking performance due to temperature were less obvious. Despite the limitations of the study in terms of sample number and the arbitrary rating system, this did provide clear evidence of what were the major factors influencing sample dissolution ethanol concentration and pH.

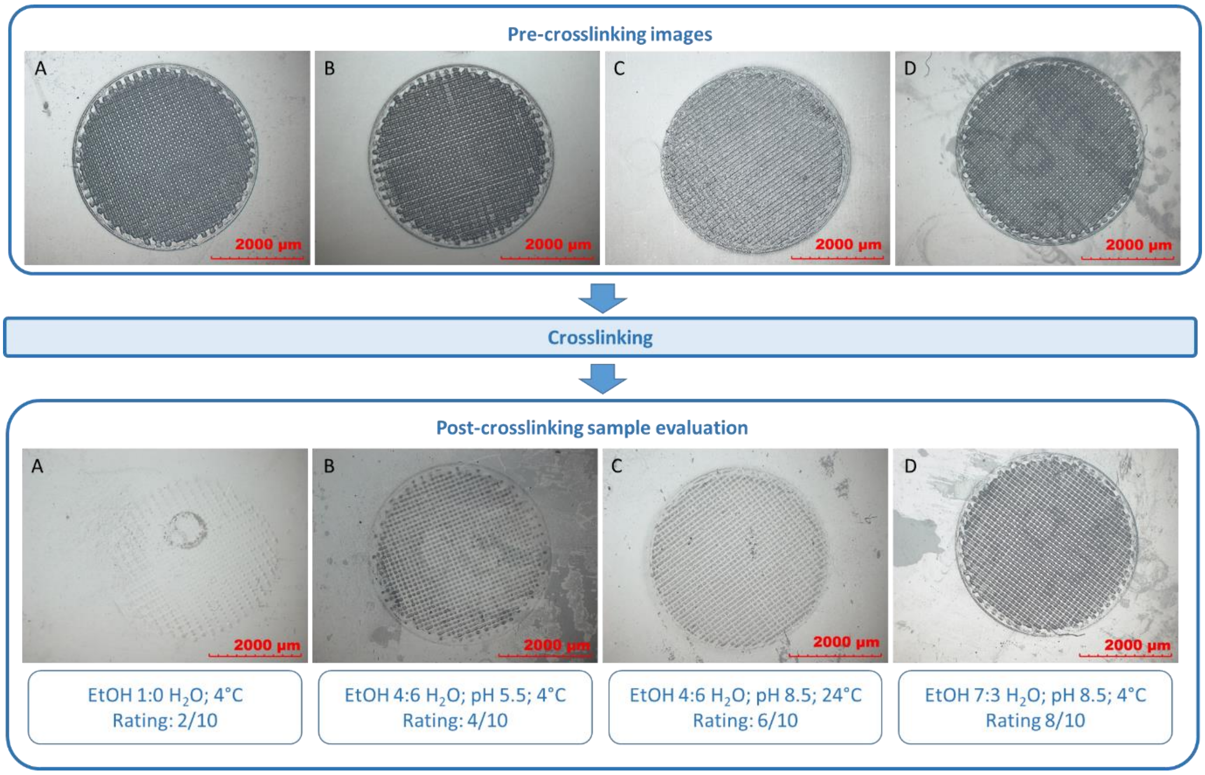

Figure S1: Images of samples A to D before and after crosslinking with the crosslinking conditions and sample rating below the post-crosslinking images of the samples. Sample A retained many superficial features, but the bulk of the samples appeared to have disappeared, most likely much collagen remained soluble and was removed during washing steps. Similarly for sample B and C there seemed to have been significant sample loss, more so for B. D appeared largely unchanged, however on closer inspection there were signs of some sample dissolution in the troughs of the print pattern.

	**pH**				
	**5.5**		**8.5**		
	**Temperature**		**Temperature**		
**Ethanol % v/v**	**4°C**	**20°C**	**4°C**	**20°C**	
**40%**	3	6	7	6	
	4	5	8	8	
**70%**	7	8	8	7	
	8	8	9	9	
**Controls**					
**pH not considered in 100% ethanol**			**pH 5.5**		
	**4°C**	**20°C**	**37°C**		
**100%**	2	2	1		
	2	4	1		

Table S2: Table of the crosslinking performance ratings.

Conclusion

A high ethanol concentration, 70% ethanol, and high pH, pH 8.5, were determined to be the most critical properties of a reaction media that would not severely disrupt sample morphology during EDC : NHS crosslinking. 4°C was chosen for further experiments as despite little evidence of its influence on sample dissolution, it would protect collagen from heat-denaturation during crosslinking.
